# The genome sequence of the pale Rhogogaster, *Rhogogaster chlorosoma *(Benson, 1943)

**DOI:** 10.12688/wellcomeopenres.18924.1

**Published:** 2023-02-06

**Authors:** Steven Falk, University of Oxford and Wytham Woods Genome Acquisition Lab, Darwin Tree of Life Barcoding collective, Wellcome Sanger Institute Tree of Life programme, Wellcome Sanger Institute Scientific Operations: DNA Pipelines collective, Tree of Life Core Informatics collective, Andrew Green, Darwin Tree of Life Consortium

**Affiliations:** 1 Independent Researcher, Kenilworth, Warwickshire, UK; 2 The Sawfly Recording Scheme, Bedford, Bedfordshire, UK

**Keywords:** Rhogogaster chlorosoma, the pale Rhogogaster, genome sequence, chromosomal, Hymenoptera

## Abstract

We present a genome assembly from an individual female *Rhogogaster chlorosoma *(the pale Rhogogaster; Arthropoda; Insecta; Hymenoptera; Tenthredinidae). The genome sequence is 255 megabases in span. The whole assembly is scaffolded into 10 chromosomal pseudomolecules. The mitochondrial genome has also been assembled and is 16.0 kilobases in length. Gene annotation of this assembly on Ensembl has identified 24,433 protein coding genes.

## Species taxonomy

Eukaryota; Metazoa; Ecdysozoa; Arthropoda; Hexapoda; Insecta; Pterygota; Neoptera; Endopterygota; Hymenoptera; Tenthredinoidea; Tenthredinidae; Tenthredininae; *Rhogogaster*; *Rhogogaster chlorosoma* (Benson, 1943) (NCBI:txid1385029).

## Background

*Rhogogaster* are green and black sawflies within the Tenthredininae subfamily. The genus is difficult to distinguish from *Tenthredo.* The genus *Rhogogaster* has around 40 species distributed across the Holarctic and Asiatic regions with the majority found in the Palaearctic region. Within this genus, numerous species clusters and subspecies have been identified. *Rhogogaster chlorosoma* and *R. scalaris* are in the *scalaris* cluster and, together with *R. punctulata* and *R. viridis*, form the *Rhogogaster* ( *Rhogogaster)* subgenus ( Liston, 1995).

Adults have been observed predating on other sawflies and flies and are not particularly associated with flowers. The larvae feed on a broad range of trees and herbs including alders, poplars, willows, rowans and meadowsweet but are not considered a pest of agricultural or horticultural crops.

The genus *Rhogogaste*r includes taxa that are difficult to distinguish morphologically, particularly within the *R. scalaris* group. Intermediate forms exist between clearly identifiable *R. scalaris* and *R. chlorosoma* suggesting either they are very close sibling species or there is introgression of alleles and traits between these taxa. Indeed, a mitochondrial CO1 barcode does not consistently separate the two species ( Taeger & Viitasaari, 2015).

The provision of a complete genome sequence for a member of this group will lay the foundation for future analyses, including facilitating comparison of nuclear gene variants from multiple individuals, and ultimately the genetic basis of the variant morphological traits. The genome sequence provided here derives from a female individual with several characters consistent with Benson’s description of *R. chlorosoma* including a lack of a black stripe along the mesopleural groove, a greater extent of green in front of the ocellar triangle and larger tarsal pulvilli ( Benson, 1952). The CO1 barcode groups closest to those from previously identified *R. chlorosoma*, although not in a monophyletic group. 

## Genome sequence report

**Figure 1.**
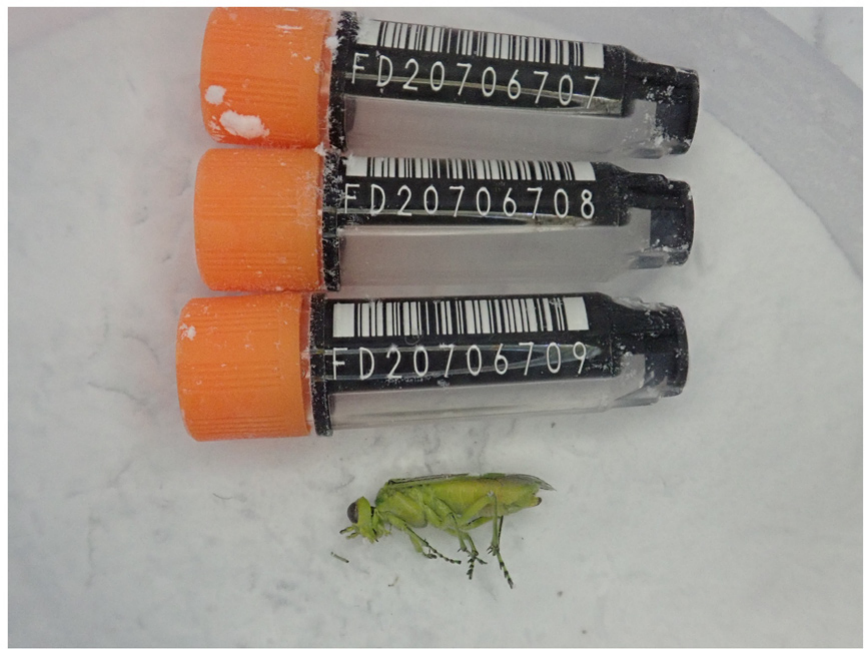
Photograph of the *Rhogogaster chlorosoma* (iyRhoChlo1) specimen used for genome sequencing.

The genome was sequenced from one female *R. chlorosoma* specimen ( Figure 1) collected from Wytham Woods (51.76, –1.33). A total of 74-fold coverage in Pacific Biosciences single-molecule HiFi long reads was generated. Primary assembly contigs were scaffolded with chromosome conformation Hi-C data. Manual assembly curation corrected five missing or mis-joins, reducing the scaffold number by 23.08% and increasing the scaffold N50 by 23.83%.

**Figure 2.**
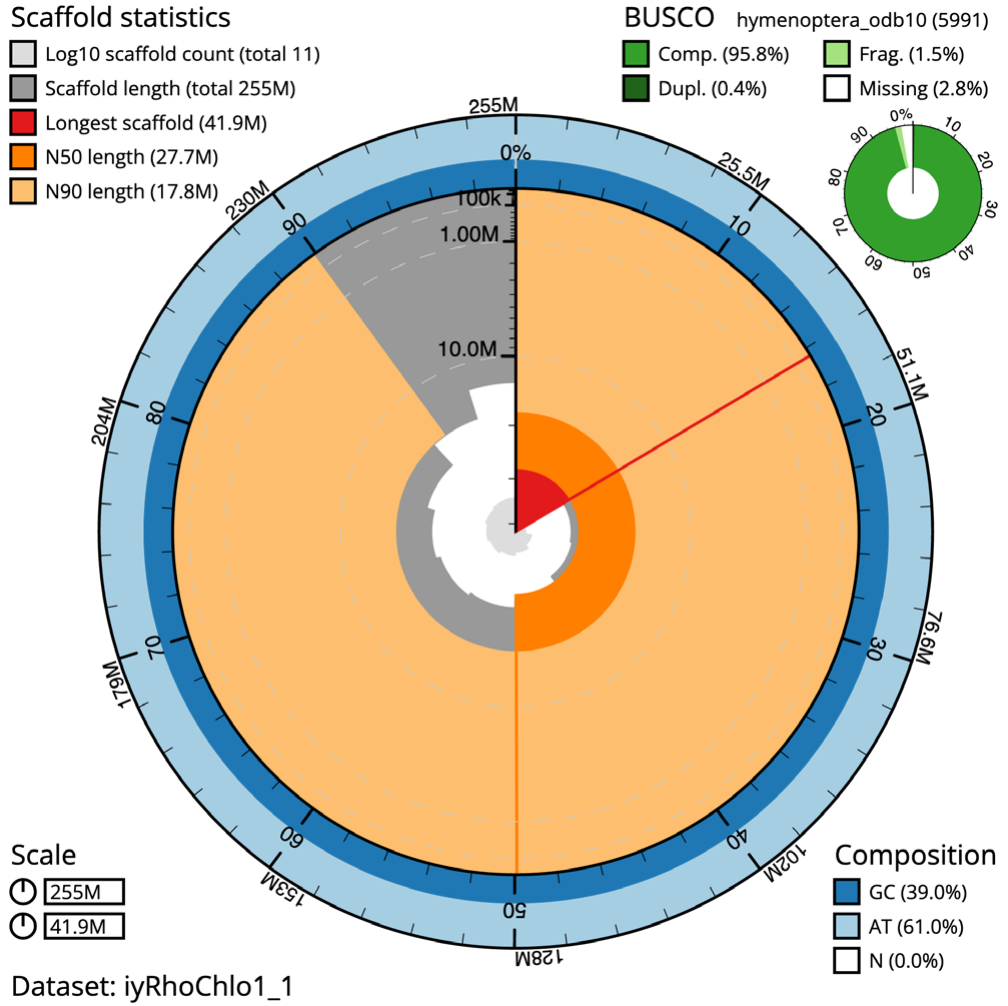
Genome assembly of *Rhogogaster chlorosoma*, iyRhoChlo1.1: metrics. The BlobToolKit Snailplot shows N50 metrics and BUSCO gene completeness. The main plot is divided into 1,000 size-ordered bins around the circumference with each bin representing 0.1% of the 255,287,193 bp assembly. The distribution of chromosome lengths is shown in dark grey with the plot radius scaled to the longest chromosome present in the assembly (41,917,220 bp, shown in red). Orange and pale-orange arcs show the N50 and N90 chromosome lengths (27,728,829 and 17,778,631 bp), respectively. The pale grey spiral shows the cumulative chromosome count on a log scale with white scale lines showing successive orders of magnitude. The blue and pale-blue area around the outside of the plot shows the distribution of GC, AT and N percentages in the same bins as the inner plot. A summary of complete, fragmented, duplicated and missing BUSCO genes in the hymenoptera_odb10 set is shown in the top right. An interactive version of this figure is available at https://blobtoolkit.genomehubs.org/view/iyRhoChlo1_1/dataset/iyRhoChlo1_1/snail.

The final assembly has a total length of 255.3 Mb in 10 sequence scaffolds with a scaffold N50 of 27.7 Mb ( Table 1). All of the assembly sequence was assigned to 10 chromosomal-level scaffolds. Chromosome-scale scaffolds confirmed by the Hi-C data are named in order of size ( Figure 2– Figure 5; Table 2). The assembly has a BUSCO v5.3.2 ( Manni *et al*., 2021) completeness of 95.8% using the hymenoptera_odb10 reference set ( *n* = 5,911). While not fully phased, the assembly deposited is of one haplotype. Contigs corresponding to the second haplotype have also been deposited.

**Table 1.** Genome data for *Rhogogaster chlorosoma*, iyRhoChlo1.1.

Project accession data		
Assembly identifier	iyRhoChlo1.1	
Species	*Rhogogaster chlorosoma*	
Specimen	iyRhoChlo1	
NCBI taxonomy ID	1385029	
BioProject	PRJEB52861	
BioSample ID	SAMEA10167069	
Isolate information	female; iyRhoChlo1: thorax (PacBio HiFi), abdomen (RNA-Seq), head (Hi-C)	
Assembly metrics*		*Benchmark*
Consensus quality (QV)	66.6	*≥ 50*
*k*-mer completeness	100%	*≥ 95%*
BUSCO**	C:95.8%[S:95.4%,D:0.4%], F:1.5%,M:2.8%,n:5991	*C ≥ 95%*
Percentage of assembly mapped to chromosomes	100%	*≥ 95%*
Sex chromosomes	N/A	*localised homologous pairs*
Organelles	Mitochondrial genome assembled	*complete single alleles*
Raw data accessions		
PacificBiosciences SEQUEL II	ERR9793197	
Hi-C Illumina	ERR9767807	
PolyA RNA-Seq Illumina	ERR10123702	
Genome assembly		
Assembly accession	GCA_944452935.1	
*Accession of alternate haplotype*	GCA_944452935.1	
Span (Mb)	255.3	
Number of contigs	27	
Contig N50 length (Mb)	12.6	
Number of scaffolds	10	
Scaffold N50 length (Mb)	27.7	
Longest scaffold (Mb)	41.9	
Genome annotation		
Number of protein-coding genes	24,433	
Number of gene transcripts	24,732	

* Assembly metric benchmarks are adapted from column VGP-2020 of “ Table 1: Proposed standards and metrics for defining genome assembly quality” from ( Rhie *et al.*, 2021). ** BUSCO scores based on the hymenoptera_odb10 BUSCO set using v5.3.2. C = complete [S = single copy, D = duplicated], F = fragmented, M = missing, n = number of orthologues in comparison. A full set of BUSCO scores is available at https://blobtoolkit.genomehubs.org/view/iyRhoChlo1_1/dataset/iyRhoChlo1_1/busco.

**Figure 3.**
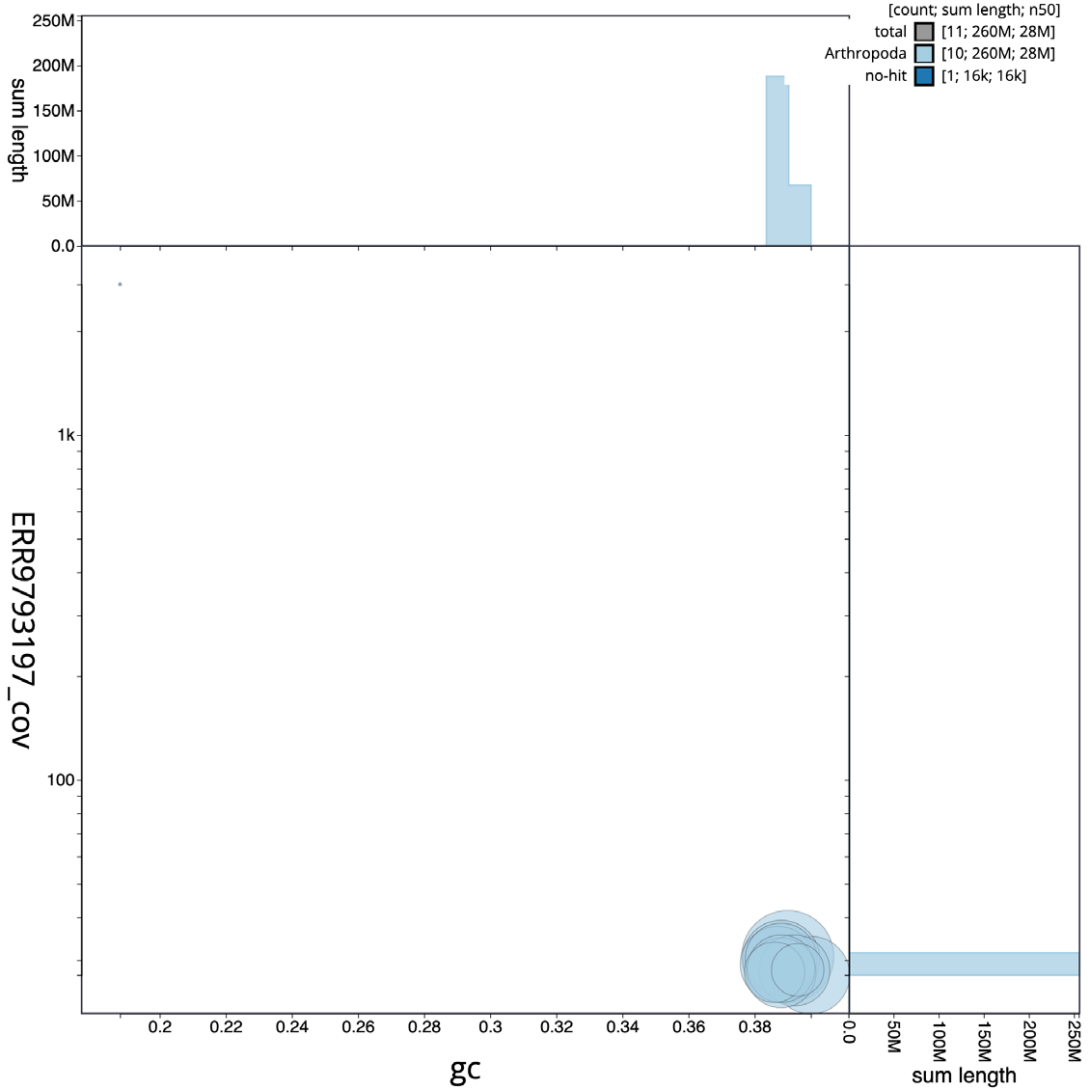
Genome assembly of *Rhogogaster chlorosoma*, iyRhoChlo1.1: GC coverage. BlobToolKit GC-coverage plot. Scaffolds are coloured by phylum. Circles are sized in proportion to scaffold length. Histograms show the distribution of scaffold length sum along each axis. An interactive version of this figure is available at https://blobtoolkit.genomehubs.org/view/iyRhoChlo1_1/dataset/iyRhoChlo1_1/blob.

**Figure 4.**
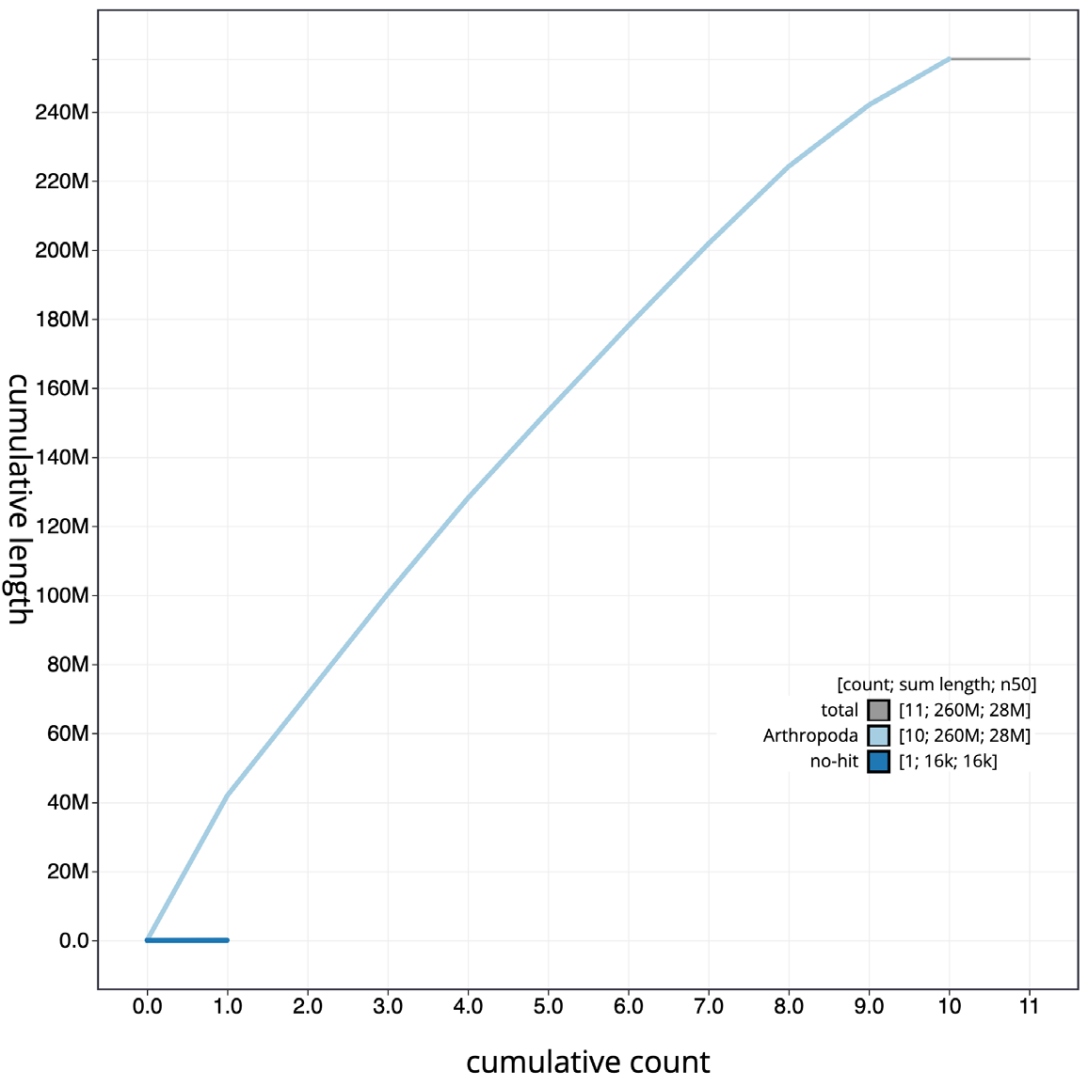
Genome assembly of *Rhogogaster chlorosoma*, iyRhoChlo1.1: cumulative sequence. BlobToolKit cumulative sequence plot. The grey line shows cumulative length for all scaffolds. Coloured lines show cumulative lengths of scaffolds assigned to each phylum using the buscogenes taxrule. An interactive version of this figure is available at https://blobtoolkit.genomehubs.org/view/iyRhoChlo1_1/dataset/iyRhoChlo1_1/cumulative.

**Figure 5.**
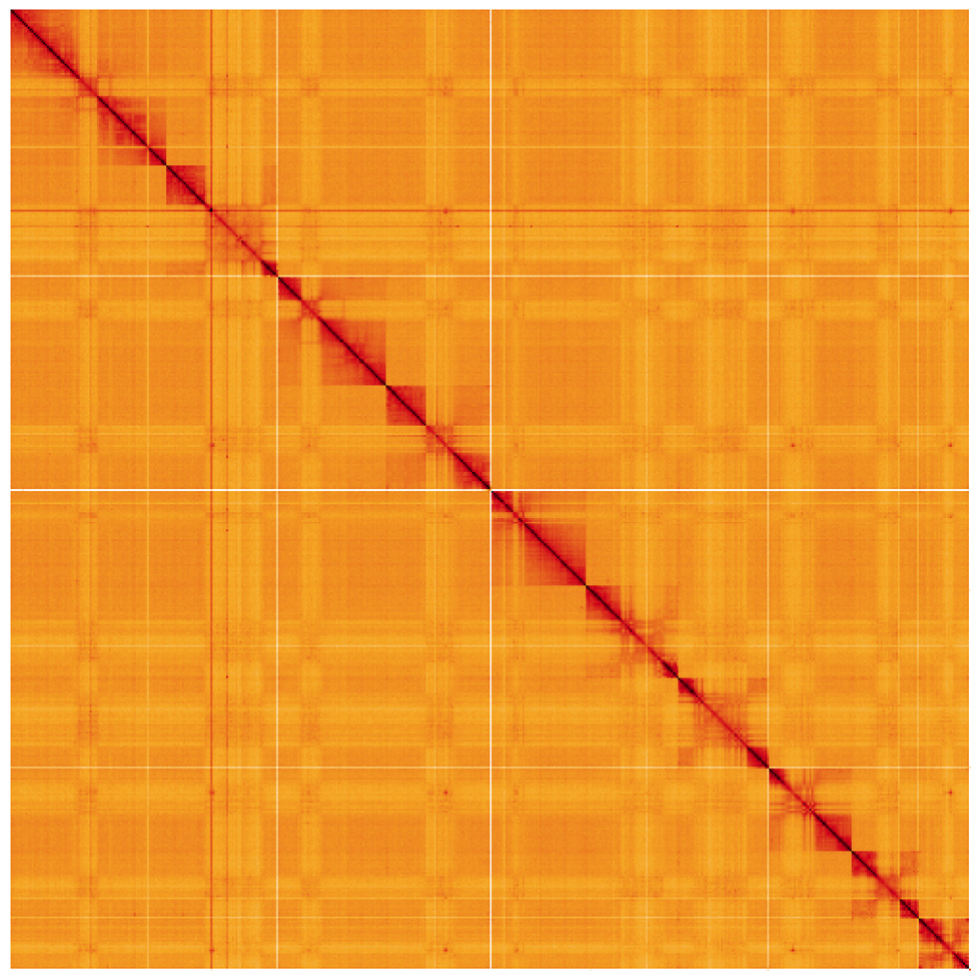
Genome assembly of *Rhogogaster chlorosoma*, iyRhoChlo1.1: Hi-C contact map. Hi-C contact map of the iyRhoChlo1.1 assembly, visualised using HiGlass. Chromosomes are shown in order of size from left to right and top to bottom. An interactive version of this figure may be viewed at https://genome-note-higlass.tol.sanger.ac.uk/l/?d=fg3tvaqYQEGht9Fg16Ks_w.

**Table 2.** Chromosomal pseudomolecules in the genome assembly of *Rhogogaster chlorosoma*, iyRhoChlo1.

INSDC accession	Chromosome	Size (Mb)	GC%
OX101797.1	1	41.92	39
OX101798.1	2	29.39	39.7
OX101799.1	3	29.04	38.8
OX101800.1	4	27.73	38.7
OX101801.1	5	25.26	38.8
OX101802.1	6	24.63	39.2
OX101803.1	7	23.8	38.8
OX101804.1	8	22.39	38.8
OX101805.1	9	17.78	38.6
OX101806.1	10	13.33	39.3
OX101807.1	MT	0.02	18.8

## Genome annotation report

The *R. chlorosoma* genome assembly GCA_944452935.1 was annotated using the Ensembl rapid annotation pipeline ( Table 1; https://rapid.ensembl.org/Rhogogaster_chlorosoma_GCA_944452935.1/). The resulting annotation includes 24,732 transcribed mRNAs from 24,433 protein-coding genes.

## Methods

### Sample acquisition and nucleic acid extraction

A female *Rhogogaster chlorosoma* specimen (iyRhoChlo1) was collected by netting in Wytham Woods, Oxfordshire (biological vice-county: Berkshire), UK (latitude 51.76, longitude –1.33) on 31 May 2021. The specimen was collected and identified by Steven Falk (independent researcher) and snap-frozen on dry ice.

DNA was extracted at the Tree of Life laboratory, Wellcome Sanger Institute (WSI). The iyRhoChlo1 sample was weighed and dissected on dry ice with tissue set aside for Hi-C sequencing. Thorax tissue was disrupted using a Nippi Powermasher fitted with a BioMasher pestle. High molecular weight (HMW) DNA was extracted using the Qiagen MagAttract HMW DNA extraction kit. HMW DNA was sheared into an average fragment size of 12–20 kb in a Megaruptor 3 system with speed setting 30. Sheared DNA was purified by solid-phase reversible immobilisation using AMPure PB beads with a 1.8X ratio of beads to sample to remove the shorter fragments and concentrate the DNA sample. The concentration of the sheared and purified DNA was assessed using a Nanodrop spectrophotometer and Qubit Fluorometer and Qubit dsDNA High Sensitivity Assay kit. Fragment size distribution was evaluated by running the sample on the FemtoPulse system.

RNA was extracted from abdomen tissue of iyRhoChlo1 in the Tree of Life Laboratory at the WSI using TRIzol, according to the manufacturer’s instructions. RNA was then eluted in 50 μl RNAse-free water and its concentration assessed using a Nanodrop spectrophotometer and Qubit Fluorometer using the Qubit RNA Broad-Range (BR) Assay kit. Analysis of the integrity of the RNA was done using Agilent RNA 6000 Pico Kit and Eukaryotic Total RNA assay.

### Sequencing

Pacific Biosciences HiFi circular consensus DNA sequencing libraries were constructed according to the manufacturers’ instructions. Poly(A) RNA-Seq libraries were constructed using the NEB Ultra II RNA Library Prep kit. DNA and RNA sequencing was performed by the Scientific Operations core at the WSI on Pacific Biosciences SEQUEL II (HiFi) and Illumina NovaSeq 6000 (RNA-Seq) instruments. Hi-C data were also generated from head tissue of iyRhoChlo1 using the Arima v2 kit and sequenced on the NovaSeq 6000 instrument.

### Genome assembly

Assembly was carried out with Hifiasm ( Cheng *et al.*, 2021) and haplotypic duplication was identified and removed with purge_dups ( Guan *et al.*, 2020). The assembly was scaffolded with Hi-C data ( Rao *et al.*, 2014) using YaHS ( Zhou *et al*., 2022). The assembly was checked for contamination as described previously ( Howe *et al.*, 2021). Manual curation was performed using HiGlass ( Kerpedjiev *et al.*, 2018) and Pretext ( Harry, 2022). The mitochondrial genome was assembled using MitoHiFi ( Uliano-Silva *et al.*, 2022), which performed annotation using MitoFinder ( Allio *et al.*, 2020). The genome was analysed and BUSCO scores were generated within the BlobToolKit environment ( Challis *et al.*, 2020). Table 3 contains a list of all software tool versions used, where relevant.

**Table 3.** Software tools and versions used.

Software tool	Version	Source
BlobToolKit	3.5.2	Challis *et al.*, 2020
Hifiasm	0.16.1-r375	Cheng *et al.*, 2021
HiGlass	1.11.6	Kerpedjiev *et al.*, 2018
MitoHiFi	2	Uliano-Silva *et al.*, 2022
PretextView	0.2	Harry, 2022
purge_dups	1.2.3	Guan *et al.*, 2020
YaHS	yahs-1.1.91eebc2	Zhou *et al.*, 2022

### Genome annotation

The BRAKER2 pipeline ( Brůna *et al.*, 2021) was used in the default protein mode to generate annotation for the *Rhogogaster chlorosoma* assembly (GCA_944452935.1) in Ensembl Rapid Release.

### Ethics/compliance issues

The materials that have contributed to this genome note have been supplied by a Darwin Tree of Life Partner. The submission of materials by a Darwin Tree of Life Partner is subject to the Darwin Tree of Life Project Sampling Code of Practice. By agreeing with and signing up to the Sampling Code of Practice, the Darwin Tree of Life Partner agrees they will meet the legal and ethical requirements and standards set out within this document in respect of all samples acquired for, and supplied to, the Darwin Tree of Life Project. Each transfer of samples is further undertaken according to a Research Collaboration Agreement or Material Transfer Agreement entered into by the Darwin Tree of Life Partner, Genome Research Limited (operating as the Wellcome Sanger Institute), and in some circumstances other Darwin Tree of Life collaborators.

## Data Availability

European Nucleotide Archive *Rhogogaster chlorosoma* (narrow-striped Rhogogaster). Accession number PRJEB52861; https://identifiers.org/ena.embl/PRJEB52861. ( Wellcome Sanger Institute, 2022) The genome sequence is released openly for reuse. The *Rhogogaster chlorosoma* genome sequencing initiative is part of the Darwin Tree of Life (DToL) project. All raw sequence data and the assembly have been deposited in INSDC databases. Raw data and assembly accession identifiers are reported in Table 1. Members of the University of Oxford and Wytham Woods Genome Acquisition Lab are listed here: https://doi.org/10.5281/zenodo.4789928. Members of the Darwin Tree of Life Barcoding collective are listed here: https://doi.org/10.5281/zenodo.4893703. Members of the Wellcome Sanger Institute Tree of Life programme are listed here: https://doi.org/10.5281/zenodo.4783585. Members of Wellcome Sanger Institute Scientific Operations: DNA Pipelines collective are listed here: https://doi.org/10.5281/zenodo.4790455. Members of the Tree of Life Core Informatics collective are listed here: https://doi.org/10.5281/zenodo.5013541. Members of the Darwin Tree of Life Consortium are listed here: https://doi.org/10.5281/zenodo.4783558.
