# Supplementary material for: The genome sequence of the pale Rhogogaster, Rhogogaster chlorosoma (Benson, 1943)
Source: Wellcome Open Res. 2023 Feb 6;8:59. [Version 1] doi: 10.12688/wellcomeopenres.18924.1 (PMC10534084; doi:10.12688/wellcomeopenres.18924.1)
Supplement: Peer review history

# Reviewer report: Reviewer response for version 1

**DOI:** 10.21956/wellcomeopenres.20982.r66072

**Published:** 2023-09-27

**Authors:** Marko Prous

**Affiliations:** 1 University of Tartu, Vanemuise, Tartu, Estonia

The article presents high quality chromosome level genome of a sawfly *Rhogogaster chlorosoma*. The methods seem to be standard ones used in Darwin Tree of Life program.

 I would be useful to address comments by a previous reviewer of the manuscript, but otherwise I did not find anything to add or correct.

Are sufficient details of methods and materials provided to allow replication by others?

Yes

Is the rationale for creating the dataset(s) clearly described?

Yes

Are the datasets clearly presented in a useable and accessible format?

Yes

Are the protocols appropriate and is the work technically sound?

Yes

Reviewer Expertise:

Sawfly taxonomy and phylogenetics.

I confirm that I have read this submission and believe that I have an appropriate level of expertise to confirm that it is of an acceptable scientific standard.

# Reviewer report: Reviewer response for version 1

**DOI:** 10.21956/wellcomeopenres.20982.r54875

**Published:** 2023-02-21

**Authors:** Craig Michell

**Affiliations:** 1 Red Sea Research Center, King Abdullah University of Science and Technology, Thuwal, Saudi Arabia

**Summary:**

 The authors here present a high-quality genome of an individual female from the species *Rhogogaster chlorosoma*, from the subfamily Tenthredininnae. The reported genome is 255,271,170 bases in length has a high contiguity and level of completeness. The genome will represent 1 out of the 27 reported Symphytan genomes to date and will provide an excellent resource for further studies into the evolution of Hymenopterans.

**Methods:**

 The authors used appropriate methods to assemble the genome of this species. The technologies used for the genome assembly include 74X genome coverage PacBio HiFi sequencing and Hi-C sequencing.

 RNAseq data was also prepared for this species, I presume for the annotation, but this is unclear in its current format.

 The genome was annotated using BREAKER2 and then input into the Ensembl rapid release pipeline.

**Datasets:**

 The datasets presented are clear and freely accessible online.

**Comments:**

 I have mostly minor comments related to making the methods more reproducible especially for people interested in performing a similar project.

- Be explicit about the length of the genome, it is only fully listed in the Figure caption for Fig2, otherwise it is rounded. Also the genome size is different to the one listed in the Ensembl Rapid release ( https://rapid.ensembl.org/Rhogogaster_chlorosoma_GCA_944452935.1/Info/Index), not by much, but a little. I presume this is likely caused by the inclusion/exclusion of N’s in the scaffolds. How many N’s are filled into your scaffolds?
- I would like to see an expansion of the methods section because at the moment it is vague and hardly reproducible.
- What was your DNA input amount for the HiFi libraries, HI-C, Transcriptomes?
- What was the average size of the HMW DNA used for the HiFi library?
- How was the Hi-C data mapped to the genome? BWA mem? Bowtie? Some other program.
- How was this data processed? How did you mark duplicates in this dataset?
- In Table 3, can you add a column with the code used to run those programs?
- What protein data was used for the annotation as BREAKER2 was run in protein mode? Did you use the BUSCO Hymenopteran protein database?
- Did you assemble the transcriptome and identify CDS? If so, how did you assemble the transcriptome and identify the CDS?

Are sufficient details of methods and materials provided to allow replication by others?

Yes

Is the rationale for creating the dataset(s) clearly described?

Yes

Are the datasets clearly presented in a useable and accessible format?

Yes

Are the protocols appropriate and is the work technically sound?

Yes

Reviewer Expertise:

My areas of expertise is in genome assembly of insects (Hymenoptera; Symphyta) and other Eukaryotes.

I confirm that I have read this submission and believe that I have an appropriate level of expertise to confirm that it is of an acceptable scientific standard.
